# Overestimation of grey matter atrophy in glioblastoma patients following radio(chemo)therapy

**DOI:** 10.1007/s10334-021-00922-3

**Published:** 2021-03-31

**Authors:** A. Gommlich, F. Raschke, J. Petr, A. Seidlitz, C. Jentsch, I. Platzek, J. van den Hoff, J. Kotzerke, B. Beuthien-Baumann, M. Baumann, M. Krause, E. G. C. Troost

**Affiliations:** 1 Siemens Energy Austria GmbH, Vienna, Austria; 2 Helmholtz-Zentrum Dresden-Rossendorf, Institute of Radiooncology – OncoRay, Dresden, Germany; 3 OncoRay - National Center for Radiation Research in Oncology, Faculty of Medicine and University Hospital Carl Gustav Carus, Technische Universität Dresden, Helmholtz-Zentrum Dresden-Rossendorf, Dresden, Germany; 4 Helmholtz-Zentrum Dresden-Rossendorf, Institute of Radiopharmaceutical Cancer Research, Dresden, Germany; 5 Department of Radiotherapy and Radiation Oncology, Faculty of Medicine and University Hospital Carl Gustav Carus, Technische Universität Dresden, Dresden, Germany; 6 Faculty of Medicine and University Hospital Carl Gustav Carus, Department of Diagnostic and Interventional Radiology, Technische Universität Dresden, Dresden, Germany; 7 Faculty of Medicine and University Hospital Carl Gustav Carus, Department of Nuclear Medicine, Technische Universität Dresden, Dresden, Germany; 8 Department of Radiology, German Cancer Research Center (DKFZ), Heidelberg, Germany; 9 German Cancer Research Center (DKFZ), Heidelberg, Germany; 10 National Center for Tumor Diseases (NCT), Partner Site Heidelberg, Germany; 11 German Cancer Consortium (DKTK), Partner Site Dresden, and German Cancer Research Center (DKFZ), Heidelberg, Germany; 12 National Center for Tumor Diseases (NCT), Partner Site Dresden, Germany: German Cancer Research Center (DKFZ), Heidelberg, Germany; Faculty of Medicine and University Hospital Carl Gustav Carus, Technische Universität Dresden, Dresden, Germany, and; Helmholtz Association / Helmholtz-Zentrum Dresden-Rossendorf (HZDR),, Dresden, Germany

**Keywords:** Radiotherapy, Tissue segmentation, SPM, Atrophy, Glioblastoma, Proton

## Abstract

**Objective:**

Brain atrophy has the potential to become a biomarker for severity of radiation-induced side-effects. Particularly brain tumour patients can show great MRI signal changes over time caused by e.g. oedema, tumour progress or necrosis. The goal of this study was to investigate if such changes affect the segmentation accuracy of normal appearing brain and thus influence longitudinal volumetric measurements.

**Materials and methods:**

T1-weighted MR images of 52 glioblastoma patients with unilateral tumours acquired before and three months after the end of radio(chemo)therapy were analysed. GM and WM volumes in the contralateral hemisphere were compared between segmenting the whole brain (full) and the contralateral hemisphere only (cl) with SPM and FSL. Relative GM and WM volumes were compared using paired t tests and correlated with the corresponding mean dose in GM and WM, respectively.

**Results:**

Mean GM atrophy was significantly higher for full segmentation compared to cl segmentation when using SPM (mean ± std: Δ*V*_GM,full_ = − 3.1% ± 3.7%, Δ*V*_GM,cl_ = − 1.6% ± 2.7%; *p* < 0.001, *d* = 0.62). GM atrophy was significantly correlated with the mean GM dose with the SPM cl segmentation (*r* = − 0.4, *p* = 0.004), FSL full segmentation (*r* = − 0.4, *p* = 0.004) and FSL cl segmentation (r = -0.35, p = 0.012) but not with the SPM full segmentation (*r* = − 0.23, *p* = 0.1).

**Conclusions:**

For accurate normal tissue volume measurements in brain tumour patients using SPM, abnormal tissue needs to be masked prior to segmentation, however, this is not necessary when using FSL.

**Supplementary Information:**

The online version contains supplementary material available at 10.1007/s10334-021-00922-3.

## Introduction

Radiotherapy is part of the standard treatment regime of gliomas. With improving survival in particular for low-grade tumours, the number of patients experiencing therapy-induced long-term complications, such as neurocognitive decline, increases accordingly [1–3]. Apart from subjective measures, such as patient-reported outcomes and neurocognitive function tests, the community strives to include objective measures in the evaluation of treatment outcome, especially when comparing different treatment modalities, such as photon- and proton-based radio(chemo)therapy.

Radiotherapy induced brain atrophy has been observed across the whole brain [4–7], hippocampus [8–11], amygdala [12] and cerebellum [13]. The findings show consistently that the amount of tissue loss is dose dependent [5, 13, 14]. Previous studies have also linked atrophy with cognitive decline [4, 15]. Brain atrophy has become an important marker of disease severity in neurodegenerative and demyelinating diseases such as Alzheimer’s disease [16] or multiple sclerosis (MS) [17]. Similarly, atrophy has the potential to become a biomarker for severity of radiation-induced side effects [4, 15]. However, this requires measurement of the normal tissue volumes of brain tumour patients with great accuracy across multiple time points. Particularly brain tumours can show great changes in MR signal intensities over time in and around the original tumour site caused by oedema, tumour progression, or treatment itself. If these signal changes influence the atrophy measurement, they could mask its potential to act as a reliable biomarker.

In the research environment, accurate measurement of atrophy commonly involves segmentation of high resolution T1-weighted MR images into grey matter (GM), white matter (WM) and cerebrospinal fluid (CSF) using software such as FSL (FMRIB Software Library: https://fsl.fmrib.ox.ac.uk/fsl/fslwiki/) [18, 19] or SPM (Statistical Parametric Mapping: https://www.fil.ion.ucl.ac.uk/spm/) [20, 21]. These software tools use an iterative Bayesian segmentation approach. By default, FSL initializes the tissue segmentation using intensity-based thresholds to segment the input image coarsely into three tissue types corresponding to GM, WM and CSF [18]. SPM uses prior tissue probability maps (TPMs) to initialize the tissue segmentation. The TPMs are derived from a collection of manually segmented healthy brains [20]. Consequently, if abnormal brain tissue is present, for instance, resection cavities, oedema, and tumour tissue in brain tumour patients, or WM lesions in MS patients, the TPMs are not valid within these regions of abnormal tissue and can cause significant bias in the resulting GM and WM segmentation. Abnormal tissue will be incorrectly classified as either GM, WM, or CSF. Consequently, image intensities of abnormal tissue contribute to the global joint-probability distribution model that is constructed for each of the GM, WM, and CSF tissues during the iterative process of segmentation. This can alter the width and position of the respective GM, WM, or CSF probability density functions on the MRI intensity scale and consequently lead to a shift between the separation of tissue boundaries, most commonly between GM/WM and GM/CSF, thus biasing volume measurements in the normal tissue even when abnormal tissue is removed after segmentation.

Segmenting MR images containing abnormal tissue has been studied previously in the field of MS, where WM lesions can have a significant impact on GM and WM volumes [22–24]. In MS, the error in volume measurements mainly arises from misclassification of WM lesions as GM or CSF [25]. Brain tumours, conversely, generally have much larger lesions. Using Bayesian segmentation approaches, this could also potentially bias normal tissue volume measurements if the abnormal tissue is not masked prior to segmentation.

The aim of this study was to assess the influence of the presence of residual tumour, resection cavities, and oedema on the quality of the tissue segmentation in the healthy tissue and on the longitudinal assessment of volumetric changes in brain parenchyma. To that end, we measured GM and WM volumes segmented from 3D 1 mm isotropic T1-weighted MR images in a cohort of glioblastoma patients with unilateral lesions. We assessed the longitudinal volumetric changes between the baseline and 3 month follow-up after radio(chemo-)therapy, respectively. We hypothesized that the presence of abnormal tissue in the ipsilateral hemisphere would affect the segmentation and longitudinal volumetric changes in the healthy contralateral hemisphere. To show that, we compared segmentations obtained from SPM and FSL with and without removing the tumour-bearing hemisphere.

## Materials and methods

### Subjects

Patient data were acquired as part of a prospective longitudinal study investigating the effect of ^11^C-methionine PET/MR for tailoring the treatment of patients with glioblastoma, approved by the local ethics committee (NCT01873469, EK41022013, BO-EK-167052020). Gross tumour resection was performed in most patients prior to radio(chemo-)therapy. Baseline MR images were acquired two to seven weeks after surgery and typically two weeks before the start of radio(chemo-)therapy. Radiotherapy was conducted using either photon or proton therapy as described previously [26]. Follow-up MRIs were acquired approximately three months after the end of radiotherapy. All patients were treated with adjuvant temozolomide [26]. More details about the patient population and treatment is provided by Seidlitz et al. [26]. Patients were excluded if no baseline MRI or no follow-up MRI was available, or if tumour or oedema extended across both hemispheres. Follow-up MRIs that showed motion artefacts or abnormal tissue in the normal appearing hemisphere were excluded, resulting in a total of 52 eligible patient datasets.

MRI data from six healthy controls (HCs) [age 39.4y ± 8.7 years, range (30.6–54.2 years), 4 male] were used to validate the data analysis strategy described in Sect. 2.3. This study was approved by the local ethics committee, and the participants gave written informed consent [DRKS-ID: DRKS00012600, EK267072017].

### Data acquisition

All MRI data were acquired on a 3 T Philips Ingenuity PET/MR scanner (Philips, Eindhoven, The Netherlands) using an eight channel head coil. For this study, pre-contrast T1-weighted (T1w) MR images were used for further analysis. In patients, T1w images were acquired in sagittal orientation using a 3D turbo field echo (TFE) sequence at 1 mm isotropic resolution (TFE inversion prepulse, TFE factor = 224, TR/TE = shortest (typical 8.2/3.8 ms), *α* = 8°, FOV 192(FH) × 224(AP) mm^2^, matrix size 192(FH) × 224(AP), 1 mm slice thickness, 192 slices). In healthy controls, T1w images were acquired using a 3D gradient spoiled echo sequence in sagittal orientation (TR = 10 ms, TE = 3.7 ms, *α* = 20°, FOV 224 × 224 mm^2^, matrix size 224 × 224, 1 mm slice thickness, 160 slices) [27].

### Data analysis

T1w images were segmented into GM, WM, CSF, bone, soft tissue and background using SPM12 [20] and GM, WM and CSF using FSL [18] with their respective default settings. SPM gave tissue probability maps as outputs, whereas FSL provided partial volume maps. For FSL, prior steps to segmentation included N4 bias field correction [28] and skull stripping [29]. The 3 T T1w images show relatively large bias field variations and without prior N4 bias field correction, FSL falsely segmented most of the subcortical GM as WM.

**Fig. 1:**
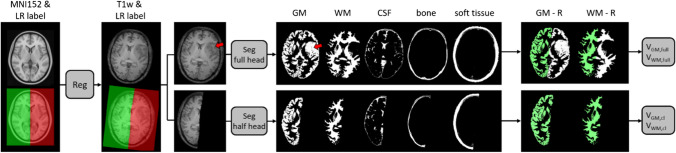
Image processing workflow: registration (Reg) of MNI152 atlas and corresponding left/right label to T1w-native space and subsequent tissue segmentation (Seg) of the full brain or contralateral (cl) hemisphere for volume assessment. In this example, abnormal tissue in the left hemisphere was mainly classified as GM (red arrow). Volume assessment was restricted to the contralateral hemisphere in all cases

GM and WM volumes of the normal appearing hemisphere were compared between segmenting the whole brain (*V*_GM,full_, *V*_WM,full_, *V*_GM + WM,full_) versus segmenting the normal appearing hemisphere (*V*_GM,cl_, *V*_WM,cl_, *V*_GM + WM,cl_). The tumour-bearing and normal appearing hemispheres were separated by non-linearly coregistering the MNI152 atlas (https://fsl.fmrib.ox.ac.uk/fsl/fslwiki/) to each T1w image using ANTs [29, 30]. The same deformation was applied to a label mask separating the two hemispheres. Subsequently, all GM and WM maps were restricted to the normal appearing hemisphere using these masks. GM and WM volumes were generated by summing all GM and WM values within these final tissue segmentation maps. Relative tissue volumes were calculated as ratios to their corresponding baseline values. The data analysis workflow is shown in Fig. 1. Mean WM and GM radiation doses in the contralateral hemisphere were calculated from the corresponding dose maps using binarized WM and GM maps, respectively, using a threshold of 0.5. The mean doses were used to compare the dose dependence of volume changes between the segmentation methods.

The comparison between the two segmentation methods (full and cl) relies on the assumption that segmenting a single hemisphere will yield the same results in that hemisphere as segmenting the whole brain, thus serving as “ground truth” for patient data with one healthy and one abnormal hemisphere. To test this assumption, the T1w images of six healthy controls were segmented. GM volumes of the left and right hemispheres obtained from whole brain segmentation were compared with those obtained from segmenting the left and right hemispheres separately.

### Statistical analysis

Relative GM, WM and GM + WM volumes of the contralateral hemispheres were compared between whole brain segmentation (full) and contralateral hemisphere segmentation (cl) using paired *t* tests. Normality of the distributions was tested using a one-sample Kolmogorov–Smirnov test. Cohen’s *d* = *μ*_*x −*_ *μ*_*y*_/*σ*_*x*–*y*_ was used to calculate the effect size.

Relative GM and WM volumes obtained from segmenting the whole brain (full) and the contralateral hemisphere (cl) were correlated with the corresponding mean dose in GM and WM of the contralateral hemisphere, respectively, using Pearson regression analysis.

## Results

Demographics of the 52 eligible patients are given in Table 1.

**Table 1:** Patients’ characteristics

Patients, *n*	52
Proton/photon, *n*	13/39
Gender (male/female), *n*	32/20
Age at start of radiotherapy (mean ± std), years	54.5 ± 14.5
Follow-up time, days	88.3 ± 12.0

GM volumes from the healthy controls show that both FSL and SPM are suitable for segmentation of a single hemisphere with very little deviation between single-hemisphere and full brain segmentation (see supplementary material section I).

**Fig. 2:**
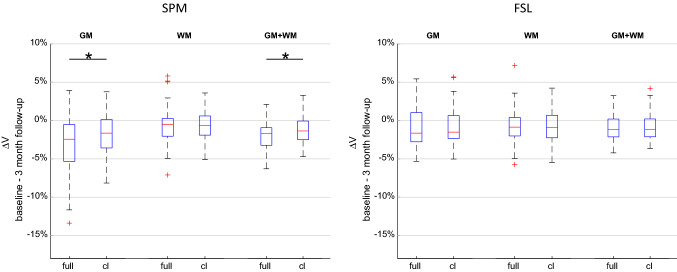
Relative volume changes of grey matter (GM), white matter (WM) and the combination of the two volumes (GM + WM) between baseline and the 3 month follow-up for SPM and FSL. Results of full brain segmentation (full) and contralateral segmentation (cl) were compared using a paired *t* test (*p < 0.05). *ΔV* change in volume

The relative GM and WM volume changes determined using full brain segmentation and contralateral hemisphere segmentation are compared in Fig. 2. With SPM, relative GM volume loss was significantly higher when using the full brain segmentation approach (mean ± std: Δ*V*_GM,full_ = − 3.1% ± 3.7%, Δ*V*_GM,cl_ = − 1.6% ± 2.7%; *p* < 0.001, *d* = 0.62). There was no significant difference in relative GM volume changes between full and cl segmentation when using FSL (mean ± std: Δ*V*_GM,full_ = − 0.9% ± 2.7%, Δ*V*_GM,cl_ = − 0.9% ± 2.5%; *p* = 0.91, *d* = 0.02). There was no significant difference in relative WM volume changes for SPM (mean ± std: Δ*V*_WM,full_ = − 0.8% ± 2.4%, Δ*V*_WM,cl_ = − 0.8% ± 2.1%; *p* = 0.95, *d* = 0.01) and FSL (mean ± std: Δ*V*_WM,full_ = − 0.75% ± 2.4%, Δ*V*_WM,cl_ = − -0.77% ± 2.2%; *p* = 0.89, *d* = 0.02).

**Fig. 3:**
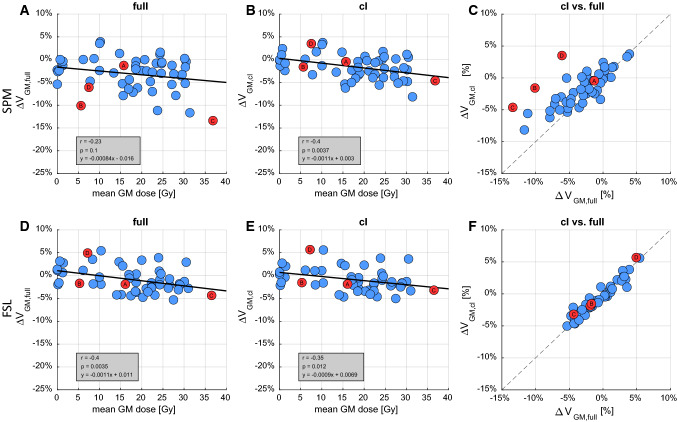
Scatter plots of relative grey matter (GM) volume changes over the mean GM dose. GM volumes were determined from SPM and FSL segmentation of full brain (**a**, **d**) and contralateral hemisphere (**b**, **e**). Regression parameters were determined using Pearson’s correlation. The volume changes determined from full brain and cl segmentation are compared in (**c**, **f**). Red markers highlight four individual examples which are shown in Fig. 4. *ΔV*_*GM*_ change in GM volume

When segmenting the full brain with SPM, relative GM volume changes (Fig. 3a) were not significantly correlated with the mean GM dose (*r* = − 0.23, *p* = 0.1). Additionally, a non-zero *x* = 0 intercept suggested GM atrophy despite a mean GM dose of 0 Gy. On the other hand, using the SPM cl, FSL full, and FSL cl segmentation, the relative GM volume changes (Fig. 3b, d, e) were significantly correlated with the mean GM dose (SPM cl: *r* = − 0.4, *p* = 0.004, FSL full: *r* = − 0.4, *p* = 0.004, FSL cl: *r* = − 0.35, *p* = 0.012) and the near zero intercepts suggest no GM changes with a mean GM dose of 0 Gy. The slopes of these three regressions were very similar, corresponding to a 1.1% (SPM cl), 1.1% (FSL full) and 0.9% (FSL cl) GM volume loss per 10 Gy mean GM dose.

**Fig. 4:**
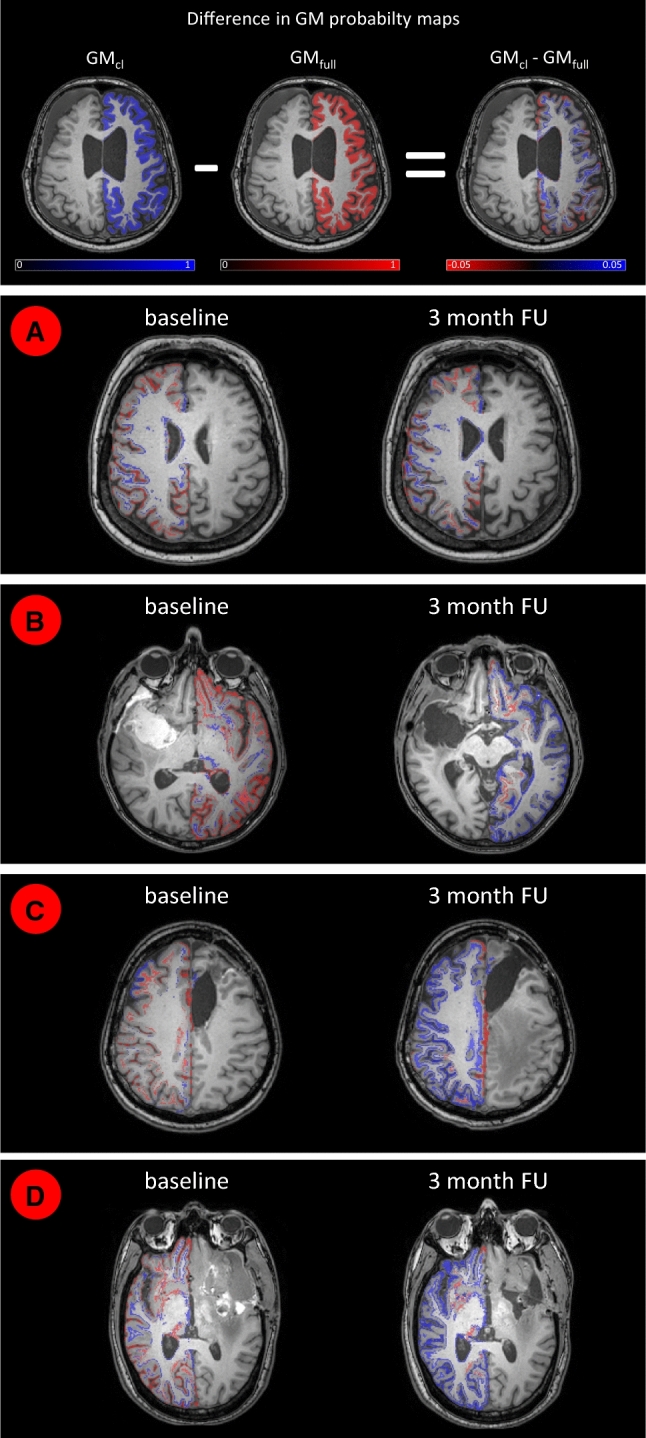
SPM segmentation: Differences of the grey matter (GM) probability maps of segmenting the contralateral hemisphere (GM_cl_, blue) minus segmenting the whole brain (GM_full_, red). Examples **a–d** are highlighted in Fig. 3. *FU* follow-up

The GM volume changes of the SPM cl (GM_cl_) and SPM full (GM_full_) segmentations are compared in Fig. 3c. While the two methods produce very similar results for some cases (e.g. highlighted example A), there are large differences for other cases (highlighted examples B, C and D). GM difference maps between SPM cl (GM_cl_) and SPM full (GM_full_) segmentation are shown for these four examples in Fig. 4. Example A shows similar relative GM volumes between full and cl segmentation in Fig. 3c and relatively consistent GM_cl_–GM_full_ difference maps (Fig. 4a). Conversely, examples B, C and D show the largest relative GM volume changes between full and cl segmentation in Fig. 3c. GM difference maps GM_c_–GM_full_ reveal these changes to be located at the GM/CSF interface around the gyri in example B (Fig. 4b), around the WM/GM interface in example C (Fig. 4c) and around both the GM/CSF and WM/GM interfaces in example D (Fig. 4d). All three examples B, C and D show pronounced MRI changes in abnormal tissue volume and contrasts between the baseline and follow-up T1w images in the ipsilateral hemispheres. Such outliers were not observed when using the FSL segmentation (Fig. 3f).

WM volume changes after 3 months were much smaller (supplementary Fig. 3), nevertheless, as for GM, WM atrophy was significantly correlated to the mean WM dose when using WM volumes obtained from SPM cl (*r* = − 0.28, *p* = 0.044), FSL full (*r* = − 0.29, *p* = 0.04) and FSL cl (*r* = − 0.41, *p* = 0.003) segmentation. The estimated degree of WM atrophy was 0.5% (SPM cl), 0.6% (FSL full) and 0.8% (FSL cl) per 10 Gy mean WM dose.

## Discussion

We have shown that GM volume measurements in the contralateral hemisphere of patients with unilateral glioblastoma differ between segmentation of the whole brain and of the contralateral hemisphere alone when using SPM. This difference is likely caused by abnormal tissue changes in the ipsilateral hemisphere, typically seen during longitudinal observations of brain tumour patients, influencing normal tissue segmentation results with SPM in the contralateral hemisphere.

In the data presented in this study, higher GM atrophy was observed when not masking the ipsilateral hemisphere containing the abnormal tissue prior to SPM segmentation (Fig. 2). Since the WM volume shows no significant change, we conclude that the remaining volume is mainly assigned to CSF. Nevertheless, there were also examples in Fig. 4c, d where underestimated GM from the SPM full segmentation was also assigned to WM. Examples B, C and D have particularly large deviations in GM volume changes between full and cl SPM segmentation (Fig. 3c) and have also shown particularly pronounced image changes in abnormal tissue volume and contrast. It is therefore likely that such large regions of MRI changes will cause greater alterations to the normal tissue probability density distributions within the iterative segmentation process and thus shift tissue separation boundaries accordingly. An additional factor is that SPM also performs simultaneous bias field correction with the tissue segmentation by default [18, 20]. Depending on regularization strength of the bias field, this will also attempt to match abnormal tissue image intensities to normal tissue intensities [31]. Therefore, the overall impact of abnormal tissue on the segmentation depends on the interaction of a number of factors and is not easily appointed to a single cause. Nevertheless, we can conclude that abnormal tissue present in the ipsilateral hemisphere of brain tumour patients will influence SPM segmentation results of the contralateral hemisphere.

Conversely, FSL segmentation results are consistent between full brain and cl segmentation. Corresponding atrophy estimates are also very similar to those obtained from SPM cl segmentation for both GM (Fig. 3) and WM (suppl. Fig. 3). Unlike SPM, by default FSL uses grey value thresholds to coarsely segment the input image into the three expected tissue types GM, WM and CSF [18]. Although the abnormal tissue has to be assigned to one of those normal tissue types as with the SPM TPM method, this seems to have no apparent impact on the segmentation results. The weakness of the threshold-based initialisation of FSL are large bias field variations. These can cause the intensity values between GM, WM and CSF to overlap [18] and that led to misclassification of subcortical GM as WM in the data presented here. However, using the established N4 bias field correction algorithm [28] prior to FSL segmentation resulted in a much improved segmentation with visually very similar results to SPM.

The findings presented here are relevant for longitudinal studies requiring accurate brain volume measurement using iterative segmentation software with TPMs for initialization. While some previous studies have considered that abnormal tissue could bias volume measurements and restricted the segmentation to the contralateral hemisphere [4, 5], others have not [7, 32, 33]. Consequently, previous findings of volume changes determined with SPM could potentially be biased [32].

A limitation of this study is the restriction of the analysis to unilateral brain tumour cases, as done in some previous studies [4, 5]. However, by co-registering individual abnormality masks for all time points of a patient into a common space, arbitrary anatomical regions can be masked as demonstrated previously [27]. This allows accurate segmentation of normal appearing tissue in cases with bilateral lesions as well, albeit spatial normalization can be difficult in patients with large mass effect [34]. Minor differences in the FSL and SPM results could be caused by the fact that SPM gave tissue probability maps as outputs, whereas FSL provided partial volume maps. However, a preliminary analysis using tissue probability maps output for FSL (“-p” option) showed no change in the overall results. Lastly, the behaviour of other segmentation tools using a similar methodology, such as AFNI [35] or Atropos [36], was not tested. However, for accurate volumes measurements, we generally suggest to exclude abnormal tissue prior to segmentation in future studies.

In conclusion, abnormal tissue present in the ipsilateral hemisphere of brain tumour patients will influence SPM segmentation results of the contralateral hemisphere. In the dataset presented here, GM atrophy was overestimated in the contralateral hemisphere when the ipsilateral hemisphere containing abnormal tissue was not removed prior to segmentation. Consequently, for accurate volume measurement in brain tumour patients using SPM, the abnormal tissue needs to be masked prior to segmentation, however, this is not necessary when using FSL.

## Supplementary Information

Below is the link to the electronic supplementary material.

Supplementary file1 (DOCX 285 KB)
