# Supplementary material for: Overestimation of grey matter atrophy in glioblastoma patients following radio(chemo)therapy
Source: MAGMA. 2021 Mar 31;35(1):145–52. doi: 10.1007/s10334-021-00922-3 (PMC8901471; doi:10.1007/s10334-021-00922-3)
Supplement: Supplementary file 1 — Supplementary file1 (DOCX 285 KB) [file 10334_2021_922_MOESM1_ESM.docx]

# Supplementary material

## Section I: ground truth


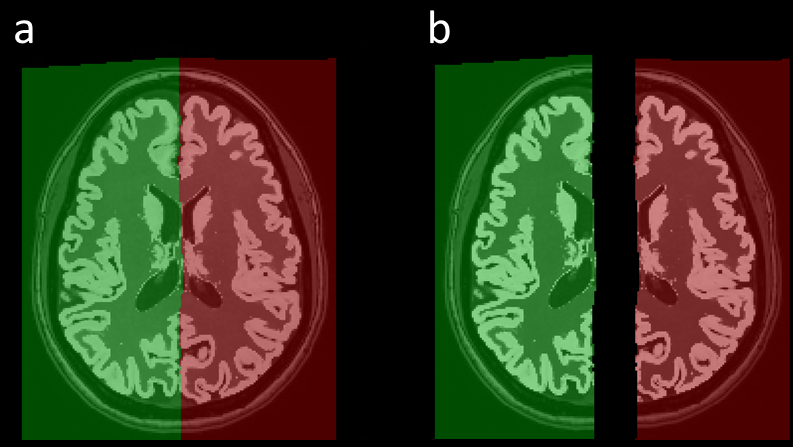


suppl. Fig 1: (a) GM reference volumes in left (red) and right (green) hemispheres determined from whole head segmentation. (b) GM test volumes from segmenting the left and right hemispheres separately.

T1w images of 6 healthy controls (HCs) were used to assess if SPM and FSL segmentation results yield similar grey matter (GM) volumes when segmenting half a brain, compared to segmenting a whole brain.

GM reference volumes $V_{GM,ref}$were calculated in the left and right hemispheres from whole brain segmentation (suppl. Fig. 1a). GM volumes from the half brain segmentation (suppl. Fig. 1b) $V_{GM,test}$ were used to calculate the deviation from the reference volumes:

$${\Delta V}_{GM,rel}=1-\frac{V_{GM,test}^{x}}{V_{GM,ref}^{x}}, x=L,R$$

This results in 12 ${\Delta V}_{GM,rel}$ values from 6 HCs and the left and right hemispheres and are shown in suppl. Fig. 2a. The mean relative GM volumes ${\Delta V}_{GM,rel}$ were 99.3% ± 0.7% (RMSE = 1%) for SPM and 100.1% ± 0.4% (RMSE = 0.4%) for FSL (suppl. Fig. 2b). Although SPM seems to consistently underestimate the GM volume when segmenting half a brain, the mean deviation is below 1%.

We therefore conclude that both SPM and FSL provide accurate segmentation results for half head segmentation and thus provide suitable ground truth GM volumes.

a

b

suppl. Fig. 2: (a) Relative GM volumes of half head segmentation (test) relative to whole head segmentation (reference). (b) Mean relative GM volumes and standard deviation determined from the individual values in (A).

## Section II: changes in white matter volume vs. dose

suppl. Fig. 3: Scatter plots of relative white matter (WM) volume changes from SPM and FSL segmentation over the mean WM dose. WM volumes were determined from segmentation of full brain (a,d) and contralateral hemisphere (b,e). Regression parameters were determined using Pearsons correlation. Abbreviation: ΔV = change in volume
